# The role of psycho-oncologic screenings in the detection and evaluation of depression in head and neck cancer aftercare patients

**DOI:** 10.1007/s00405-021-07017-8

**Published:** 2021-08-18

**Authors:** Stefan Hadas, Maximilian Huhn, Michael Rentrop, Barbara Wollenberg, Stephanie Combs, Steffi Pigorsch, Anja Pickhard, Anna Maria Stefanie Buchberger

**Affiliations:** 1 Hals-Nasen-Ohren Klinik und Poliklinik, Klinikum Rechts der Isar, Technischen Universität München, Ismaningerstr. 22, 81675 München, Deutschland; 2 Klinik und Poliklinik für Psychiatrie und Psychotherapie, Klinikum Rechts der Isar, Technischen Universität München, Ismaningerstr. 22, 81675 München, Deutschland; 3 Klinik für Strahlentherapie und Radioonkologie, Klinikum Rechts der Isar, Technischen Universität München, Ismaningerstr. 22, 81675 München, Deutschland; 4 Klinik für Strahlentherapie, Universitätsklinikum Augsburg, Stenglinstr. 2, 86156 Augsburg, Deutschland; 5 Klinik für Psychiatrie und Psychotherapie, Sozialstiftung Bamberg, Klinikum Bamberg, Buger Straße 80, 96049 Bamberg, Deutschland; 6 Klinik für Psychiatrie und Psychotherapie, Kbo-Inn-Salzach-Klinikum, Gabersee-Str 7, 83512 Wasserburg Am Inn, Deutschland

**Keywords:** Depression, EORTC, Head and neck cancer aftercare, M.I.N.I., Psycho-oncological screening, WHO-5

## Abstract

**Purpose:**

Cancer and morbidity during a therapeutic regimen can result in somatic and psychiatric impairment. We have evaluated the need of appropriate psychological screening by analyzing a large collective of head and neck cancer (HNC) patients with particularly burdensome symptoms.

**Methods:**

HNC-aftercare patients were asked about somatic and psychological symptoms by means of standardized questionnaires of the European Organization for Research and Treatment of Cancer (EORTC Q30 and QLQ-H&N35). Patients with poor well-being values on the World Health Organization-5-Well-Being Index were screened for depression by using the Mini International Neuropsychiatric Interview, and adequate treatment was initiated, if necessary.

**Results:**

Our sample consisted of 453 HNC-aftercare patients (average age 64.5 years; 72.0% male; 28.0% female). 25.1% showed abnormalities based on their WHO-5 questionnaire. A current major depressive episode was observed in 8.5% of the total study group. Patients with lip and oral cavity tumors showed the highest depression prevalence (18.9%). Time since initial HNC diagnosis showed no clear trend with regard to the number of depression cases. 50.0% of patients with a current major depressive episode consented to receiving assistance and/or therapy. Within the total study population, the most burdensome symptoms were found to be “dry mouth” (48.3%), “trouble doing strenuous activities” (46.0%), “trouble taking a long walk” (38.5%), and “worry” (35.5%). Aftercare patients with a depression diagnosis tended to have heavier symptom burdens than people without major depression.

**Conclusions:**

Despite the various cancer-related burdensome factors, prevalence levels of depression among the HNC-aftercare patients and the general population were similar. Nevertheless, since the number of diagnosed depression cases is high, the need for psychological treatment should be considered within the tumor collective. Furthermore, screening for depression should be implemented in clinical routines by using the appropriate standardized questionnaires.

**Supplementary Information:**

The online version contains supplementary material available at 10.1007/s00405-021-07017-8.

## Background

More than 18 million new cancer cases were detected worldwide in 2018 [1]. Related burdensome symptoms [2] can often be fulminant and need to be dealt with promptly to improve patient well-being. Head and neck cancer (HNC) is associated with visibly noticeable specific stress-causing factors. Typical tumor- or therapy-associated symptoms in everyday clinical practice include visible scars, prostheses, restrictions of identity-forming language, swallowing problems, therapy-resistant pain, and diffuse fear caused by uncertainty about the future. These factors can clearly strongly influence the psyche of the affected patients.

Many patients with HNC show an association with alcohol and tobacco, especially when these factors are combined [3, 4]. Alcohol consumption itself can increase the risk of depression [5]. Moreover, stigmatization, for example because of a visible loss of physical integrity, can affect the state of mood of the patient [6]. These factors should therefore be taken into account to estimate a patient’s risk for developing depression [7].

Depression is a severe psychological disease that often has serious symptoms [7, p. 81 ff.]. Hematological, gynecological, and lung cancer patients have been identified to present a higher risk of depression than other tumor patients [8]. Nevertheless, studies have indicated that, on summarizing a variety of different tumor entities, only slight differences can be observed in depression prevalence between tumor patients and the general population [9]. Internationally, the 12-month prevalence of depression in tumor patients is 9% [9]. Moreover, a risk of overlooking depressive states exists with regard to patients having tumor entities that are less associated with depression hazard factors, because of insufficient focus on their psychic symptoms.

In spite of the increasing number of certified ear, nose, and throat (ENT)-HNC centers in Germany, no standardized psycho-oncological screening and co-supervision supplemental to somatic treatment has been established. However, appropriate sensitization concerning depression is crucial, given the high rates of depression [9, 10, 11] and HNC prevalence [11, p. 21, 57 ff.] and the possible severe consequences of a depressive episode [7, p. 81 ff., 12]. With regard to HNC aftercare, very little data has been collected concerning the number of depression cases and possible somatic triggers. Therefore, we have analyzed the general status of well-being and symptoms of a large study population of 453 ENT-HNC-aftercare patients.

## Methods

In this retrospective study, we analyzed the results of standardized patient questionnaires that were distributed to patients from 2016 to 2017 (9.5 months) during a weekly ENT-HNC-aftercare consultation appointment (at the ENT Department of a German university hospital with a certified HNC center). No exclusion criteria for specific tumor entities, therapy options, or aftercare periods were established to present conclusive results for the entire cross section group of HNC-aftercare patients. All persons who filled out the screening questionnaires were included (the screening questionnaire was usually completed autonomously before seeing the doctor). This retrospective anonymized study was conducted with the approval of the local ethics committee (136/18 S).

### Statistical methods

The data were analyzed using the SPSS statistical analysis program (version 24) [13]. Since our data were based on descriptive statistics, frequency tables and cross-tabulations were created to show frequencies in general and those of the selected subgroups (e.g., age and gender). To enable further interpretation, we compared the data with previously published frequency data. All of our analyses refer to valid percent values. The analyses of possible relationships between symptom burdens and depression diagnosis were limited to the ten most often stated common complaints (all complaints listed in Table 6). In this context and for the statistical tests with regard to tumor entities as well as time since the first HNC diagnosis, the “no depression group” was defined as individuals having no current major depression episode based on the Mini International Neuropsychiatric Interview (M.I.N.I.) or a WHO-5 sum score of ≥ 13.

Time since first diagnosis only included initial HNC, with no other tumor entities being taken into account. Initial diagnosis was defined as the date of the (rigid) panendoscopy or first verifiable therapy. Here, we always chose the first of the month. If only a year was given, we chose the month of June (first of the month).

The data that support the findings of this study are available on request from the corresponding author.

#### Initial evaluation of the quality of life and of somatic and psychic well-being

The set of questionnaires consisted of the World Health Organizations’ WHO-5 questionnaire [14, p. 25 Annex 1, 15], further specific questions that were created by the study designers, and the quality of life questionnaires of the European Organization for Research and Treatment of Cancer (EORTC) QLQ-C30 (core questionnaire) and -H&N35 (HNC-module) [16, 17]. The scores of the WHO-5 items, with a scale of 0 (worst value) to 5 (best value) in each case, were summed [14, p. 25 Annex 1] (the items refer to the last 2 weeks [15]). Additionally, patients were set specific questions concerning previous treatments and medication. In the EORTC questionnaires, items 1–28 of QLQ-C30 and 31–60 of QLQ-H&N35 were included, because of their identical and therefore comparable answer scales assigning the level of stress/complaints for each question in a range of 1 (“not at all”) to 4 (“very much”) [17]. Items 1–5 did not refer to a specific time period, whereas all other items referred to the last week [17]. In this study, the two highest answers, namely (3) “quite a bit” and (4) “very much”, were added up for each question and sorted in descending order.

#### Implemented diagnostic tool for depression (Mini International Neuropsychiatric Interview (M.I.N.I.)) and therapy initiation

If the WHO-5 sum score was < 13 [14, p. 25 Annex 1], a specific standardized questionnaire was used to validate depression. For this purpose, the A module of M.I.N.I. [18, 19] was used in the setting of a private interview between a trained health professional and the patient, provided that the patient was in agreement. The diagnostic interview was conducted according to the instructions for use [19]. The findings formed the basis for the support and treatment measures that were offered to the patients and initialized with their consent.

## Results

### Composition of the study group and study design

**Fig. 1:**
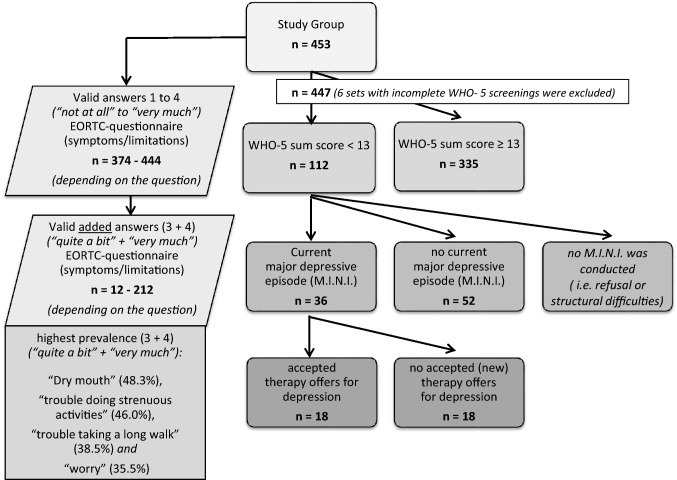
Consort flow diagram: EORTC answers (symptoms/limitations) and mood disorders. This graph was designed using Microsoft PowerPoint 2010: © 2010 Microsoft Corporation

453 sets of questionnaires (≙ 453 patients) were distributed to ENT-HNC-aftercare patients during a 9.5-month period. Descriptive statistics for additional detailed information about the composition of the study group in general and of their HNC diagnoses are provided in Tables 1 and 2. The general study design is shown in the consort diagram (Fig. 1) with an overview of the number of EORTC answers (somatic complaints) and the number of mood disorders and therapy. All of the following results refer to the available valid percent values.

**Table 1:** General Information of the study group

Study group	453 patients
Gender distribution	Male: (♂): 326 patients (72.0%) Female: (♀): 127 patients (28.0%)
Average age	64.54 years
Age distribution	≤ 40 years: 8 (1.8%); ♂: 4 (50.0%), ♀: 4 (50.0%) 41–60 years: 151 (33.3%); ♂: 108 (71.5%), ♀: 43 (28.5%) ≥ 61 years: 294 (64.9%); ♂: 214 (72.8%), ♀: 80 (27.2%)

**Table 2:** Tumor entities of the study group

Tumor entities^a^		
	Frequency	Valid percent
*Valid*		
Oropharynx	132	29.1
Larynx	112	24.7
Lip and oral cavity	41	9.1
Hypopharynx	37	8.2
Skin tumors (i.e., spinalioma, basal cell carcinoma, melanoma, Bowen’s disease)	30	6.6
Multiple tumor locations/sites, synchronous/combined tumors	25	5.5
Nasal cavity and paranasal sinuses	21	4.6
Major salivary glands	18	4.0
Carcinoma of unknown primary (CUP)	17	3.8
Nasopharynx	8	1.8
Thyroid gland	5	1.1
*Others*	*7*	*1.5*
Total	453	100.0

^a^Only ENT patients (no consideration of secondary tumors or previous tumors in other medical specialties)

### Analysis of the WHO-5 questionnaires

Of the distributed 453 sets of questionnaires (≙ 453 patients), 9 sets of the WHO-5 questionnaires were not completely answered. Nevertheless, three of these had a sum score ≥ 13 and thus remained in the study group. The remaining six sets with incomplete screenings were excluded from the analyses referring to the WHO-5 sum score. Thus, a total of 447 questionnaires were analyzed (≙447 patients). Of these, 25.1% (112 patients) had a WHO-5 sum score below the cutoff of < 13 (Table 3a). The results categorized into subgroups regarding age and gender are listed in Table 3ba–dc. Especially for distinct age groups, differences in the prevalence of a rather poor state of “well-being”, suggested by a WHO-5 sum score < 13, were apparent. The age group of “41–60 years” showed a prevalence of a WHO-5 sum score < 13 of 33.6%, and the age group “ ≥ 61 years” showed 21.4% compared to 0% in the small age group of “below 40 years” (N ≤ 40 years = 8).

**Table 3:** Number of patients with a WHO-5 sum score of < 13 and current major depressive episode

		*WHO-5 sum score of < 13*					
Study group	*N*	YES *N*	%	NO *N*	%		
(a) Total study group (with valid values)	*447*	*112*	25.1%	*335*	74.9%		
(ba) ≤ 40 years old	*8*	*0*	0.0%	*8*	100.0%		
(bb) 41–60 years old	*149*	*50*	33.6%	*99*	66.4%		
(bc) ≥ 61 years old	*290*	*62*	21.4%	*228*	78.6%		
(ca) ≤ 40 years old ♂	*4*	*0*	0.0%	*4*	100.0%		
(cb) 41–60 years old ♂	*106*	*33*	31.1%	*73*	68.9%		
(cc) ≥ 61 years old ♂	*211*	*42*	19.9%	*169*	80.1%		
(da) ≤ 40 years old ♀	*4*	*0*	0.0%	*4*	100.0%		
(db) 41–60 years old ♀	*43*	*17*	39.5%	*26*	60.5%		
(dc) ≥ 61 years old ♀	*79*	*20*	25.3%	*59*	74.7%		

		*Current major depressive episode*					
Study group	*N*	YES *N*	%	NO *N*	%	No M.I.N.I *N*	%
(e) Total study group (with valid values)	*423*	*36*	8.5%	*52*	12.3%	*335*	79.2%
(fa) ♂	*307*	*26*	8.5%	*35*	11.4%	*246*	80.1%
(fb) ♀	*116*	*10*	8.6%	*17*	14.7%	*89*	76.7%
(g) Diagnostic interview using M.I.N.I (if the WHO-5 sum score is < 13)	*88*	*36*	40.9%	*52*	59.1%		
(ha) Diagnostic interview using M.I.N.I (if the WHO-5 sum score is < 13) ♂	*61*	*26*	42.6%	*35*	57.4%		
(hb) Diagnostic interview using M.I.N.I (if the WHO-5 sum score is < 13) ♀	*27*	*10*	37.0%	*17*	63.0%		

*N* Number of patients with valid values

Exclusion criteria: (a to hb): patients with uncompleted WHO-5 questionnaires, if the sum score was still not ≥ 13 (≙ cutoff-score)

and/or (e to hb): patients without M.I.N.I. in spite of having a WHO-5 sum score of < 13

### Analysis of the M.I.N.I. interview for patients with WHO-5 sum score below cutoff

The diagnostic interview (M.I.N.I.) for clarification/screening for the possible depression was subsequently offered to these 112 patients with an evidently pathological WHO-5 sum score of < 13. No interview data were collectable from 24 of these 112 patients because of structural difficulties (i.e., the patient had left the consultation before implementation of the questionnaire or the patient did not wish to be interviewed). Thus, the M.I.N.I interview was carried out with 88 aftercare patients.

40.9% (36 patients) of the 88 interviewed ENT-HNC-aftercare patients were subsequently diagnosed with a “current major depressive episode” by using the M.I.N.I. (Table 3g). Distribution by gender in this subgroup was unequal between female (37.0%) and male (42.6%) patients, as can be seen in Table 3ha–hb.

In relation to the total study group with regard to the 423 available valid values, we diagnosed a current depression episode in 8.5% of all patients (Table 3e) with an almost equal gender distribution (Table 3fa–fb) of depression diagnoses in women (8.6%) and men (8.5%).

In this subgroup of 36 patients with current depression, a total of 52.8% (19 patients) were also found to have had a “prior major depressive episode” (with a follow-up time period without mood deficiency of at least 2 months [19]) (Supplement).

Information concerning “current and/or past depression treatment” was available only for 31 of these 36 patients, revealing that 45.2% (14 patients) were under current treatment or had had past treatment for a major depressive disorder.

#### Analysis of the depression prevalence regarding tumor entities as well as time point of diagnosis

We found differing percentages of “major depression episode” diagnosed by M.I.N.I. among patients with regard to their specific tumor entities as shown in Table 4. Especially patients with tumors of the lips and oral cavity (18.9%) as well as the subgroup summarizing skin tumors (11.1%) were showing the highest depression prevalence as shown in Table 4.

**Table 4:** Current major depressive episode depending on tumor entities

Current major depressive episode^a,b^					
Tumor entities^c^			Frequency (N)	Percent	Valid percent
Pharynx	Valid	Yes	16	9.0	9.7
		No^d^	149	84.2	90.3
		Total	165	93.2	100.0
	Missing		12	6.8	
	Total	177	100.0		
Larynx	Valid	Yes	6	5.4	5.6
		No^d^	101	90.2	94.4
		Total	107	95.5	100.0
	Missing		5	4.5	
	Total	112	100.0		
Lip and oral cavity	Valid	Yes	7	17.1	18.9
		No^d^	30	73.2	81.1
		Total	37	90.2	100.0
	Missing		4	9.8	
	Total	41	100.0		
Skin tumors (i.e., spinalioma, basal cell carcinoma melanoma, Bowen’s disease)	Valid	Yes	3	10.0	11.1
		No^d^	24	80.0	88.9
		Total	27	90.0	100.0
	Missing		3	10.0	
	Total	30	100.0		
Synchronous/combined tumors, multiple tumors locations/sites	Valid	No^d^	22	88.0	100.0
	Missing		3	12.0	
	Total	25	100.0		
Nasal cavity and paranasal sinuses	Valid	Yes	2	9.5	9.5
		No^d^	19	90.5	90.5
		Total	21	100.0	100.0
Major salivary glands	Valid	No^d^	16	88.9	100.0
	Missing		2	11.1	
	Total	18	100.0		
Carcinoma of unknown primary (CUP)	Valid	Yes	1	5.9	6.3
		No^d^	15	88.2	93.8
		Total	16	94.1	100.0
	Missing		1	5.9	
	Total	17	100.0		
Thyroid gland	Valid	No^d^	5	100.0	100.0
Others	Valid	Yes	1	14.3	14.3
		No^d^	6	85.7	85.7
		Total	7	100.0	100.0

^a^Study population: total study group *N* = 453; total of missing values: 30; *N* (valid percent) = 423

^b^The underlying questions refer to the last 2 weeks

^c^Only ENT patients (no consideration of secondary tumors or previous tumors in other medical specialties)

^d^No current major depression episode (M.I.N.I.) or WHO-5 sum score of ≥ 13

**Table 5:** Connection between first HNC diagnosis and depression prevalence

Current major depressive episode * Time (full months) since first diagnosis of HNC cross-tabulation^a^									
			Time (full months) since first diagnosis of HNC^b,c,d^						
			≤ 12	13—24	25—36	37—48	49—60	≥ 61	Total
Current	Yes	Count	6	5	4	5	2	14	36
Major depressive episode^e^		% within current major depressive episode	16.7%	13.9%	11.1%	13.9%	5.6%	38.9%	100.0%
	No^f^	Count	59	55	31	36	33	164	378
		% within current major depressive episode	15.6%	14.6%	8.2%	9.5%	8.7%	43.4%	100.0%
Total		Count	65	60	35	41	35	178	414
		% within current major depressive episode	15.7%	14.5%	8.5%	9.9%	8.5%	43.0%	100.0%

^a^Test population: total study group (valid values)

^b^Only initial diagnoses of HNC were taken into account (no other tumor entities)

^c^Time between initial diagnosis of HNC and questionnaire

^d^Initial diagnose of HNC: panendoscopy or first verifiable therapy; we always chose the first of the month;

if only a year was given, we chose the month of June (first of the month)

^e^The underlying questions refer to the last 2 weeks

^f^No current major depression episode (M.I.N.I.) or WHO-5 sum score of ≥ 13

The times since initial HNC diagnosis varied between 3 months and a little more than 41 years, with a mean follow-up of 5.6 years. No clear trend was apparent between the diagnosis of depression and the time interval of the first HNC diagnosis with an almost equal distribution of patients (16.7–11.1%) with diagnosed depression for the first 4 years of the initial 5-year tumor follow-up (Table 5). For the fifth year of tumor aftercare, the number of depression diagnosis dropped to 5.6% (Table 5). But we want to point out that 38.9% of identified patients with a depression episode presented above the standard 5-year follow-up margin (Table 5).

#### Therapeutic approach for patients with pathologic M.I.N.I.

**Fig. 2:**
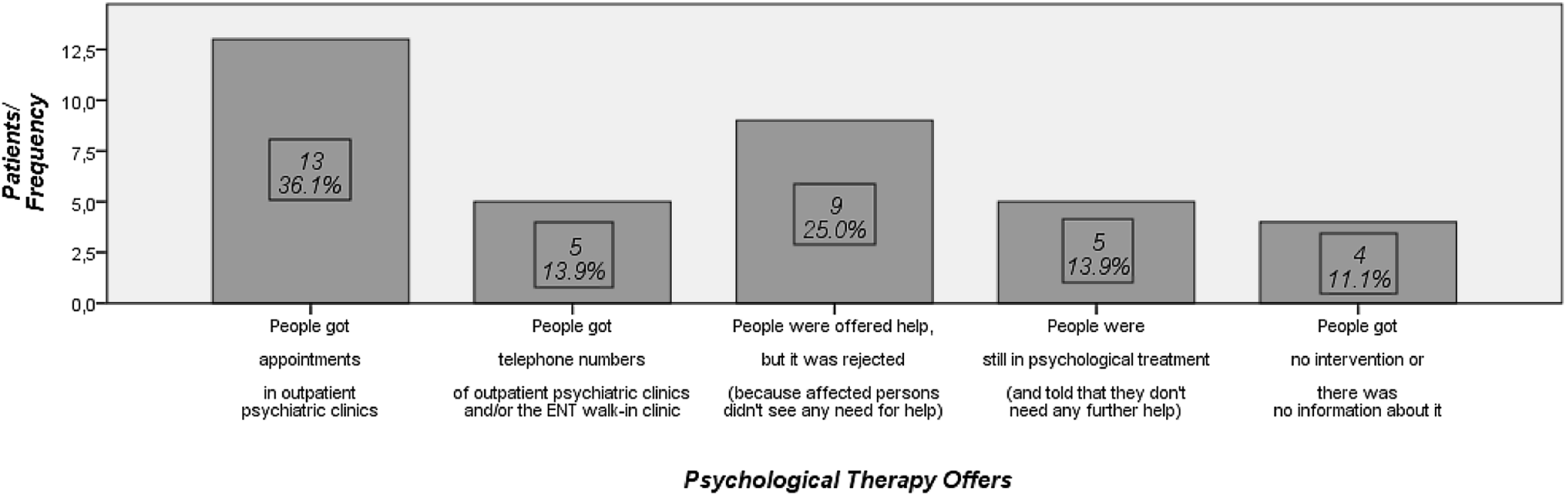
Offers of therapy (subgroup with “current major depressive episode “). Test population: all patients with “current major depressive episode” (using M.I.N.I.); *N* = 36; the underlying items refer to the last 2 weeks. Please note: prescriptions of psychiatric drugs are not shown in the figure above. Please note: if a patient was given an appointment in an outpatient psychiatric clinic, he/she was not listed again in one of the other categories (no double-counting). This graph was designed using SPSS Statistics (Version 24): Licensed Materials – Property of IBM Corp. © Copyright IBM® Corporation and its licensors 1989, 2016

Figure 2 summarizes the therapy offered to the aftercare patients diagnosed with “current major depressive episode” by using M.I.N.I. 36.1% (13 patients) received immediate appointments in outpatient psychiatric clinics. 13.9% (5 patients) preferred to call the outpatient psychiatric clinic and/or the ENT walk-in clinic themselves to arrange a meeting or decided to take the telephone numbers of the clinics in case of an emergency caused by mood deterioration. Patients without a diagnosed episode of depression sometimes also asked for help, since they were encouraged by the additional attention provided to deal with these matters. Of these patients, 13.5% confirmed one of the two options (made an appointment in outpatient psychiatric clinic or took the telephone numbers).

#### Analysis of the EORTC QLQ-C30 and QLQ-H&N35 questionnaires

The results of the EORTC questionnaire relating to the symptoms/limitations are shown in Table 6. The different number of cases is attributable to the (unintentional or intentional) unanswered items in the questionnaire. “Dry mouth” (48.3%), “trouble doing strenuous activities” (46.0%), “trouble taking a long walk” (38.5%), and “worry” (35.5%) had the highest prevalence in our study population (Table 6).

**Table 6:** Symptoms/limitations of ENT-HNC-aftercare patients

Items/complaints (Percentage frequencies of the answers; “quite a bit “ and “very much “ were added)	Ranking	*N(3* + *4)*	*N(1 to 4)*
Dry mouth (48.3%)	1	212	439
Trouble doing strenuous activities* (46.0%)	2	202	439
Trouble taking a long walk* (38.5%)	3	170	441
Worry (35.5%)	4	156	440
Less sexual enjoyment (35.5%)	4	133	374
Sticky saliva (35.3%)	6	152	431
Cough (35.0%)	7	154	440
Less interest in sex (33.4%)	8	129	386
Felt tired (32.9%)	9	144	438
Felt weak (31.9%)	10	141	442
Problems with sense of taste (31.6%)	11	137	434
Trouble sleeping (31.5%)	12	139	441
Problems swallowing solid food (31.4%)	13	137	437
Need to rest (30.7%)	14	135	439
Limited in pursuing hobbies or other leisure time activities (27.9%)	15	122	437
Trouble eating (27.9%)	15	121	434
Problems with teeth (27.6%)	17	120	434
Limited in doing either work or other daily activities (27.4%)	18	118	430
Short of breath (26.8%)	19	116	434
Trouble talking on telephone (26.5%)	20	116	438
Interference of social activities because of physical condition or medical treatment (25.6%)	21	113	442
Problems with sense of smell (25.3%)	22	110	435
Pain (24.9%)	23	110	442
Tension (24.7%)	24	109	440
Felt depressed (24.2%)	25	106	437
Hoarseness (24.0%)	26	103	429
Trouble enjoying meals (23.6%)	27	103	437
Irritability (22.5%)	28	98	435
Interference of daily activities because of pain (22.5%)	28	98	436
Trouble talking to other people (22.4%)	30	98	436
Felt ill (21.3%)	31	94	440
Problems in opening the mouth wide (21.0%)	32	92	438
Financial difficulties because of physical condition or medical treatment (20.3%)	33	89	438
Interference of family life because of physical condition or medical treatment (19.6%)	34	85	434
Trouble eating in front of other people (18.1%)	35	79	437
Difficulty remembering things (17.8%)	36	77	433
Appetite loss (17.1%)	37	75	440
Choking when swallowing (17.0%)	38	74	435
Painful throat (15.9%)	39	69	435
Pain in mouth (15.8%)	40	69	437
Appearance bothers you (15.6%)	41	69	440
Trouble going out in public (15.5%)	42	68	439
Need to stay in bed or chair during the day* (14.5%)	43	63	435
Pain in jaw (13.5%)	44	59	436
Difficulty in concentrating on things (13.3%)	45	59	444
Trouble taking a short walk outside the house* (13.0%)	46	57	437
Problems swallowing liquids (13.0%)	46	57	440
Trouble eating in front of family (12.4%)	48	54	436
Constipation (11.8%)	49	52	441
Problems swallowing pureed food (9.9%)	50	43	434
Trouble having social contact with friends (9.4%)	51	41	438
Trouble having social contact with family (8.9%)	52	39	438
Trouble having physical contact with family or friends (8.7%)	53	37	427
Soreness in mouth (8.2%)	54	35	430
Nausea (6.8%)	55	30	440
Diarrhea (6.1%)	56	27	443
Need help with eating, dressing, washing, or using the toilet* (4.0%)	57	18	442
Vomiting (2.8%)	58	12	441

*N (1 to 4)* = *total number of all patients with a valid answer 1 to 4 (“not at all”/”a little”/”quite a bit”/”very much”)*

*N (3* + *4)* = *total number of all patients with a valid answer 3 to 4 (”quite a bit”/”very much”)*

^*^Items do not refer to a specific time period; all other items refer to the last week

#### Correlation of the EORTC QLQ-C30 QLQ-H&N35 questionnaires and M.I.N.I

Cross-tabulations between the ten most frequent complaints and patients with or without a current major depression episodes showed the following results: for all analyzed questions, HNC-aftercare patients with a diagnosis of depression showed a higher percentage of the general symptom burden answers “very much” and “quite a bit” than people without such diagnosis, with only one exception: namely the percentage “quite a bit” of “trouble taking a long walk” was 0.3% higher in the no-depression group, but the answer “very much” was, as previously mentioned, higher in the depression group (38.9% vs. 11.9%).

Depending on the question, the percentage frequencies were 3.5–40.4% (answer “very much”) or 3.4–31.5% (answer “quite a bit”) higher in patients with a current major depression episode.

Nevertheless, patients without depression also had a considerable degree of symptom burden.

The detailed data are shown in the supplement.

## Discussion

A large cross-sectional sample of 453 ENT-HNC-aftercare patients was retrospectively analyzed with regard to their physical and mental complaints by using a specific combination of validated questionnaires consisting of the WHO-5, EORTC QLQ-C30 and -H&N35 and structured M.I.N.I. interview.

### Choice of questionnaires

The WHO-5 well-being questionnaire is suited for an initial screening of the general mood [20], since patients can answer the items autonomously in less than 5 min while waiting for their appointment, because of the form’s comprehensibility and conciseness. This standardized questionnaire has been validated for over 30 languages [20] and allows the health professional accurately to check the mental status of the patient and decide within seconds if a further exploration of the patient’s psychological well-being might be needed. For a further evaluation, the depressive disorder module of M.I.N.I. allows a relatively quick as well as valid and reliable assessment of major depressive episodes because of its modular and well-structured composition [21, 22]. Since a health professional is nevertheless needed for the interview, the requirements for additional work cannot be denied. However, in our opinion, the additional gain in the health and contentment of the patient from using these measures outweighs the extra effort needed.

Validated EORTC questionnaires are suitable for comprehensively establishing the symptoms and complaints suffered by HNC patients [23], but the time needed by the patient to fill them out and then by the medical staff to analyze them makes such standardized forms unsuitable for quick screening under the time management needed during consultation appointments. We did, however, analyze the data retrospectively to gain an insight into the connection between specific symptoms, general symptom burden, and status of mood. Additionally, in our experience the questionnaire helped patients to reflect upon their symptoms and burden of symptoms preparing for the doctor–patient conversation.

### WHO-5 screening

About a quarter of all screened patients of our study group had a WHO-5 sum score below the cutoff of 13 points indicating a rather poor state of well-being at the time of taking the questionnaire. Depending on the age group, this percentage varied. Our findings suggest that particularly younger patients between 41 and 60 years are affected by subdued moods. Our data showed a higher percentage of 33.6% for the age group “41–60 years”, while the group “ ≥ 61 years” revealed 21.4% with a WHO-5 sum score < 13. Brähler et al. published for the general German population depending on these age groups that 15% of the group “41–60-year-old”, respectively, 19% of the group “ ≥ 61 year-old” disclosed a WHO-5 sum score < 13 [24]. However, in the age subgroup of patients up to 40 years, none of our patients had a score of < 13 in contrast to the standard population value of 13% reported by Brähler et al. [24]. Since this age subgroup was only compounded by eight patients in our study group, a statistical comparison to the general population seems insignificant.

Split into groups by gender, the percentage of people with a WHO-5 sum score of < 13 (inside the subgroups by age with an adequate number of patients: 41–60 years and ≥ 61 years) also exceeded the comparable values, whereby in each case, the greatest percentage differences were again seen in the age group 41–60 years [24]. This finding speaks against general age or gender as strong influencer of a reduced state of well-being as one might assume based on the comparable numbers in the general population. It accentuates the direct and indirect effects of tumor disease and morbidity in concerns of not only somatic but also mental health.

### M.I.N.I. interview: depression prevalence, correlations and therapeutic approach

#### Prevalence of depression

During the assessment period of 9.5 months, 8.5% (36 patients) of the cross-sectional ENT-HNC-aftercare patients were diagnosed with a “current major depressive episode” based on the M.I.N.I., comparable to the findings about the depression prevalence of HNC patients of Rohde et al. of 9.3% [25].

Of 88 patients who were screened with M.I.N.I. because of their WHO-5 sum score < 13, 40.9% (36 patients) had a “current major depressive episode” and 52.8% (19 patients) of these 36 diagnosed individuals stated to have been diagnosed before (“previous major depressive episode”), indicating that a one-time depression screening is not enough.

A wide range of presented depression prevalence can be found in topic-related reviews (e.g., 0–38% for cancer patients in general [26] and 15–50% for HNC patient [27]) depending on the method of data collection and time of observation. The comparative data for Germany indicates the prevalence of a 12-month major depressive disorder among cancer patients of various medical specialties of 8.0% (including recurrent episodes) or 12.5%, respectively (also including “mood disorder due to a medical condition”) [28]. International studies indicate a 12-month depression prevalence of 9% among patients with various kinds of cancer [9]. Surprisingly, the German general population shows a similar 12-month major depressive disorder prevalence of 8.3% [29] and 6.8% [10] (unipolar depression 8.2%) [10]. In a study by Vehling et al., many cancer patients suffered from diverse psychiatric complaints (anxiety and/or affective disorder), in spite of a similar prevalence for depressive disorders compared with the general population [9]. Moreover, the authors mentioned that further psycho-social burdens were observed that might not have been properly grouped by the commonly established classifications [9]. Linden et al. have shown that 11.1% of HNC patients have depression symptoms in the clinical range, but this is lower than, for example, the value of 17.9% in patients with lung tumors [8]. Therefore, in concordance with our data, the depression prevalence of patients with HNC seems to be closer to the value of the general population than that for some other cancer entities.

#### Correlation of gender, time of diagnosis and tumor site with depression

In the general population, women tend to higher numbers of diagnosed depression [10]. In a study by Linden et al., women with HNC as well as other tumor entities were more affected by depression than men [8]. In our study, contrary to these findings, the depression prevalence of men (42.6%) was slightly above the values for women (37.0%) in the subgroup with diagnostic interview using M.I.N.I., or they showed no meaningful differences when correlated to the overall study group. Therefore, our findings indicate the need to screen for depressive disorders, regardless of gender distribution.

In our study, the time between HNC diagnosis/period of tumor aftercare and questionnaire had no strong influence regarding the likelihood of a depression diagnosis. But our data indicate different rates of depression depending on the type of tumor. While Rohde et al. have described the highest prevalence of an episode of major depression (28.5%) for laryngeal cancer [25], in our study, the highest prevalence rates were found in tumors related to the lips and oral cavity (18.9%) compared to only 5.6% of patients with laryngeal carcinoma. People with major salivary gland tumors, thyroid gland tumors, and multiple/ synchronous tumors had the lowest prevalence with 0.0% in each, although the small number of patients in these subgroups makes them difficult to compare with the higher numbered tumor entities. The other tumor entities lay in between these values (e.g., skin tumors (11.1%), tumors of the pharynx (9.7%) or nasal cavity and paranasal sinuses (9.5%)).

The various number of cases of major depression within our study suggest that diagnosed depression is at least partially dependent on the type of tumor. Nevertheless, the variable results (e.g., those regarding laryngeal carcinoma prevalence) demonstrate that depression not only depends on the location of the tumor, but also on other factors. Therefore, major depression screening should be standard for all ENT-HNC-aftercare patients, regardless of gender, age, type of tumor or time of the first HNC diagnosis.

#### Therapeutic approach

Because of the variety of partly long-lasting symptoms, specific psychological care should be offered during both acute treatment [30] and long-term tumor aftercare. But screening for depression is only significant if help and treatment options can be offered. In our study subgroup with a diagnosis of depressive disorder, 50.0% accepted immediate, non-pharmaceutical intervention. But pharmaceutical treatment is another option [31, p. 66 ff.]. As prescriptions provided by a psychiatrist or general practitioner were, in part, not evaluable, information concerning the pharmacological treatment of depression in our patients was not included in this study and the estimated number of patients who had sought/were seeking treatment after diagnoses was most likely much higher than 50.0%. Interestingly, 45.2% in this subgroup declared themselves to be under current and/or past therapy. These results lead to the conclusion that, even when a patient is under current and/or past specific depression treatments, frequent re-clarification of their current state of well-being is necessary. It should then be considered whether a new cycle of treatment has to be administered because of a possible relapse, or whether the ongoing therapy should be optimized.

### EORTC QLQ-C30 and QLQ-H&N35 questionnaires: symptoms and symptom burden

Many factors could have contributed to the decreased state of well-being of our cancer patients (~ 25% < 13 points in WHO-5 Screening). HNC, in particular, can cause a variety of extremely impairing symptoms. Most of the reported symptoms by the EORTC questionnaire possibly resulted from direct or indirect (partly long-lasting/persistent) effects of the tumor disease or morbidity due to the therapy [31, 32].

When comparing the ten most stressful symptoms in patients with and without depression, a clear tendency is seen toward a stronger symptom burden in patients with a diagnosed depression. This illustrates not only the importance of a detailed registration and alleviation of somatic complaints, but also stresses the concomitant evaluation of depression, especially in cases of severe symptoms. As advised by the German national psycho-oncology guidelines, adequate treatment of physical symptoms can also be important for mental health [32, p. 36]. Thus, therapy, for example pain treatments [33], not only fulfills the main aim of improving somatic symptoms, but also contributes to improvements in the mental condition of the patient [32, p. 36].

Regardless of their causality, the high incidence of impairments listed by our study group (with and without a diagnosis of depression) has to be considered. The mentioned symptoms and complaints, which might greatly influenced the quality of life individually or in combination, should therefore be generally taken into consideration when conducting tumor aftercare. Nevertheless, because of the variability of the presented symptoms, the specific complaints of each patient have to be considered on an individual basis. Overall, the results presented in this study indicate the strong demands of ENT-HNC-aftercare patients for somatic (e.g., symptom relief) and psychological support.

### Patient acceptance of this set of questionnaires

In general, the additional screening and diagnostics concerning symptoms, well-being and the psychological evaluation were positively accepted. Tumor aftercare took place as usual, and the handing out of systematized forms was merely an additional element. Whenever the questionnaire values made further discussion necessary, patients were given more time with the medical staff.

Some patients who were not diagnosed with an acute/active depressive episode as revealed by M.I.N.I. nevertheless asked for psycho-oncological/ psychiatric assistance. Thus, we conclude that this screening is an efficient addition which of course does not replace the doctor–patient conversation, but helps to structure it and emphasize certain topics of importance for both doctor and patient.

## Study limitations

One limitation of the study is the dependency of these questionnaires on the honesty and cooperation of patients. Since the WHO-5 screening was administered prior to the doctor’s appointments, a negative influence attributable to a certain degree of anxiousness about the following tumor aftercare seems possible.

Since M.I.N.I. was performed after the doctor was consulted and after the general examination as well as the (ultrasound) imaging of the neck, possible “worrying news” might have influenced the answers of the patients. However, this seems unlikely, because of the structured and precise items within the questionnaire asking about the state of well-being of the patient during the total time period of the last 2 weeks.

Very few patients refused to answer the questionnaires, making bias concerning the selection of patients in a more psychologically burdened group unlikely, especially since our rate of detected depression is comparable to the general population.

Furthermore, those patients with a sum score of ≥ 13 and “0 or 1” answers for the single-answer items were not screened for depression, as suggested by the WHO [14, p. 25 Annex 1]. In addition, we cannot rule out that, in rare cases, patients with a WHO-5 sum score of ≥ 13 also suffer from depression. Thus, the genuine overall number of patients with an acute episode of depression might be higher than in our study. Moreover, these screening instruments did not register other psychological disorders, e.g., anxiety disorders that might also occur in tumor patients.

## Conclusion

### Clinical implications

To our knowledge, this is the first study to examine a large sample of 453 ENT-HNC-aftercare patients with regard to their physical and mental complaints by using this specific set of questionnaires (WHO-5, EORTC QLQ-C30 and -H&N35, M.I.N.I.).

Numerous serious symptoms seen in the aftercare of cancer patients require individual treatments; this applies to patients with or without a diagnosis of depression. Nevertheless, patients with depression have a particularly high symptom burden. In spite of the many tumor-related stressful factors, similar depression prevalence rates between the general population and the entire ENT-HNC-aftercare collective should not lead to an underestimation of mental treatment demands of tumor patients. Because of the high prevalence rates, a psychological examination is indicated for all tumor aftercare patients independent of age, sex, ENT tumor entity, time period since first HNC diagnosis, or (previous/acute) treatment for depressive disorders and (pre-existing/acute) depressive episodes. Screening for mental health problems can easily be integrated into the clinical routine by using questionnaires (e.g., WHO-5 and M.I.N.I.) combined with doctor–patient conversations. The subsequent validation of depression among patients with abnormal WHO-5-screening results by using M.I.N.I. indicates that more than 40% of these patients truly suffer from a “current major depressive episode.” Comprehensive tumor aftercare aims to improve the quality of life of patients by alleviating their physical and mental problems. Determination of the depressive episodes and the offer of adequate treatment considerably improve the quality of life of patients. Moreover, severe consequences caused by depressive episodes, e.g., suicide, can be prevented.

## Supplementary Information

Below is the link to the electronic supplementary material.

Supplementary file1 (DOCX 109 KB)

## Data Availability

Data are available from the corresponding author upon request.

## Code availability

d.n.a.
