# Supplementary material for: The role of psycho-oncologic screenings in the detection and evaluation of depression in head and neck cancer aftercare patients
Source: Eur Arch Otorhinolaryngol. 2021 Aug 18;279(4):2143–56. doi: 10.1007/s00405-021-07017-8 (PMC8930863; doi:10.1007/s00405-021-07017-8)
Supplement: Supplementary file 1 — Supplementary file1 (DOCX 109 KB) [file 405_2021_7017_MOESM1_ESM.docx]

# Supplemental Information

| **Gender distribution^a^** | | | | |
| --- | --- | --- | --- | --- |
|  | | Frequency | Valid percent |  |
| Valid | **Male** | 326 | 72.0 |  |
|  | **Female** | 127 | 28.0 |  |
|  | Total | 453 | 100.0 |  |
| a. Test population: **total study group** | | | | |

| **Mean age^a,b^** | | | | | |
| --- | --- | --- | --- | --- | --- |
|  | N | Minimum | Maximum | **Mean** | Std. deviation |
| **Age** | 453 | 24 | 91 | 64.54 | 10.604 |
| Valid N (listwise) | 453 |  |  |  |  |
| a. Test population: **total study group** | | | | | |
| b. At the time of the questionnaire screening/interview | | | | | |

| **Age and gender distribution^a,b^** | | | | |  |
| --- | --- | --- | --- | --- | --- |
| **Age (years)** | | | Frequency | Valid percent | |
| **≤ 40** | Valid | **Male** | 4 | 50.0 | |
|  |  | **Female** | 4 | 50.0 | |
|  |  | Total | 8 | 100.0 | |
| **41–60** | Valid | **Male** | 108 | 71.5 | |
|  |  | **Female** | 43 | 28.5 | |
|  |  | Total | 151 | 100.0 | |
| **≥ 61** | Valid | **Male** | 214 | 72.8 | |
|  |  | **Female** | 80 | 27.2 | |
|  |  | Total | 294 | 100.0 | |
| a. Test population: **total study group** | | | | |  |
| b. At the time of the questionnaire screening/interview | | | | |  |

| **Age category^a,b^** | | | | |
| --- | --- | --- | --- | --- |
|  | | Frequency | Valid percent | Cumulative percent |
| Valid | **≤ 40 years** | 8 | 1.8 | 1.8 |
|  | **41–60 years** | 151 | 33.3 | 35.1 |
|  | **≥ 61 years** | 294 | 64.9 | 100.0 |
|  | Total | 453 | 100.0 |  |
| a. Test population: **total study group** | | | | |
| b. At the time of the questionnaire screening/interview | | | | |

| **WHO-5 sum score of < 13^a,b,c^** | | | | |
| --- | --- | --- | --- | --- |
|  | | Frequency | Percent | Valid percent |
| Valid | **Yes (0–12)** | 112 | 24.7 | 25.1 |
|  | **No (13–25)** | 335 | 74.0 | 74.9 |
|  | Total | 447 | 98.7 | 100.0 |
| Missing |  | 6 | 1.3 |  |
| Total | | 453 | 100.0 |  |
| a. Test population: **total study group** | | | | |
| b. Only fully completed questionnaires (444 patients) or uncompleted questionnaires with a WHO-5 sum score of ≥ 13 (3 patients) were included | | | | |
| c. The WHO-5 questions refer to the last 2 weeks | | | | |

| **WHO-5 sum score of < 13^a,b,c^** | | | | | |
| --- | --- | --- | --- | --- | --- |
| **Age (years)** | | | Frequency | Percent | Valid percent |
| **≤ 40** | Valid | **No (13–25)** | 8 | 100.0 | 100.0 |
| **41 - 60** | Valid | **Yes (0–12)** | 50 | 33.1 | 33.6 |
|  |  | **No (13–25)** | 99 | 65.6 | 66.4 |
|  |  | Total | 149 | 98.7 | 100.0 |
|  | Missing |  | 2 | 1.3 |  |
|  | Total | | 151 | 100.0 |  |
| **≥ 61** | Valid | **Yes (0–12)** | 62 | 21.1 | 21.4 |
|  |  | **No (13–25)** | 228 | 77.6 | 78.6 |
|  |  | Total | 290 | 98.6 | 100.0 |
|  | Missing |  | 4 | 1.4 |  |
|  | Total | | 294 | 100.0 |  |
| a. Test population: **total study group** | | | | | |
| b. Only fully completed questionnaires (444 patients) or uncompleted questionnaires with a WHO-5 sum score of ≥ 13 (3 patients) were included | | | | | |
| c. The WHO-5 questions refer to the last 2 weeks | | | | | |

| **WHO-5 sum score of < 13^a,b,c^** | | | | | | |
| --- | --- | --- | --- | --- | --- | --- |
| **Age (years)** | **Gender** | | | Frequency | Percent | Valid percent |
| **≤ 40** | **Male** | Valid | **No (13–25)** | 4 | 100.0 | 100.0 |
|  | **Female** | Valid | **No (13–25)** | 4 | 100.0 | 100.0 |
| **41–60** | **Male** | Valid | **Yes (0–12)** | 33 | 30.6 | 31.1 |
|  |  |  | **No (13–25)** | 73 | 67.6 | 68.9 |
|  |  |  | Total | 106 | 98.1 | 100.0 |
|  |  | Missing |  | 2 | 1.9 |  |
|  |  | Total | | 108 | 100.0 |  |
|  | **Female** | Valid | **Yes (0–12)** | 17 | 39.5 | 39.5 |
|  |  |  | **No (13–25)** | 26 | 60.5 | 60.5 |
|  |  |  | Total | 43 | 100.0 | 100.0 |
| **≥ 61** | **Male** | Valid | **Yes (0–12)** | 42 | 19.6 | 19.9 |
|  |  |  | **No (13–25)** | 169 | 79.0 | 80.1 |
|  |  |  | Total | 211 | 98.6 | 100.0 |
|  |  | Missing |  | 3 | 1.4 |  |
|  |  | Total | | 214 | 100.0 |  |
|  | **Female** | Valid | **Yes (0–12)** | 20 | 25.0 | 25.3 |
|  |  |  | **No (13–25)** | 59 | 73.8 | 74.7 |
|  |  |  | Total | 79 | 98.8 | 100.0 |
|  |  | Missing |  | 1 | 1.3 |  |
|  |  | Total | | 80 | 100.0 |  |
| a. Test population: **total study group** | | | | | | |
| b. Only fully completed questionnaires (444 patients) or uncompleted questionnaires with a  WHO-5 sum score of ≥ 13 (3 patients) were included | | | | | | |
| c. The WHO-5 questions refer to the last 2 weeks | | | | | | |

| **Current major depressive episode^a,b^** | | | | |
| --- | --- | --- | --- | --- |
|  | | Frequency | Percent | Valid percent |
| Valid | **Yes** | 36 | 7.9 | 8.5 |
|  | **No** | 52 | 11.5 | 12.3 |
|  | **No M.I.N.I.^c^** | 335 | 74.0 | 79.2 |
|  | Total | 423 | 93.4 | 100.0 |
| Missing |  | 30 | 6.6 |  |
| Total | | 453 | 100.0 |  |
| a. Test population: **total study group** | | | | |
| b. The underlying questions refer to the last 2 weeks | | | | |
| c. WHO-5 sum score of ≥ 13 | | | | |

| **Current major depressive episode^a,b^** | | | | | |
| --- | --- | --- | --- | --- | --- |
| **Gender** | | | Frequency | Percent | Valid percent |
| **Male** | Valid | **Yes** | 26 | 8.0 | 8.5 |
|  |  | **No** | 35 | 10.7 | 11.4 |
|  |  | **No M.I.N.I.^c^** | 246 | 75.5 | 80.1 |
|  |  | Total | 307 | 94.2 | 100.0 |
|  | Missing |  | 19 | 5.8 |  |
|  | Total | | 326 | 100.0 |  |
| **Female** | Valid | **Yes** | 10 | 7.9 | 8.6 |
|  |  | **No** | 17 | 13.4 | 14.7 |
|  |  | **No M.I.N.I.^c^** | 89 | 70.1 | 76.7 |
|  |  | Total | 116 | 91.3 | 100.0 |
|  | Missing |  | 11 | 8.7 |  |
|  | Total | | 127 | 100.0 |  |
| a. Test population: **total study group** | | | | | |
| b. The underlying questions refer to the last 2 weeks | | | | | |
| c. WHO-5 sum score of ≥ 13 | | | | | |

| **Current major depressive episode^a,b^** | | | |
| --- | --- | --- | --- |
|  | | Frequency | Valid percent |
| Valid | **Yes** | 36 | 40.9 |
|  | **No** | 52 | 59.1 |
|  | Total | 88 | 100.0 |
| a. Test population: **all patients who were interviewed using the M.I.N.I. (major depression module)** | | | |
| b. The underlying questions refer to the last 2 weeks | | | |

| **Current major depressive episode^a,b^** | | | | |  |
| --- | --- | --- | --- | --- | --- |
| **Gender** | | | Frequency | Valid percent | |
| **Male** | Valid | **Yes** | 26 | 42.6 | |
|  |  | **No** | 35 | 57.4 | |
|  |  | Total | 61 | 100.0 | |
| **Female** | Valid | **Yes** | 10 | 37.0 | |
|  |  | **No** | 17 | 63.0 | |
|  |  | Total | 27 | 100.0 | |
| a. Test population: **all patients who were interviewed using the M.I.N.I. (major depression module)** | | | | |  |
| b. The underlying questions refer to the last 2 weeks | | | | |  |

| **Previous major depressive episode^a,b^** | | | | |
| --- | --- | --- | --- | --- |
|  | | Frequency | Valid percent |  |
| Valid | **Yes** | 19 | 52.8 |  |
|  | **No** | 17 | 47.2 |  |
|  | Total | 36 | 100.0 |  |
| a. Test population: **all patients with "current major depressive episode" based on the M.I.N.I.**  (the underlying questions refer to the last 2 weeks) | | | | |
| b. The patients need to have at least 2 months without depressive episode/loss of interest after the last depressive episode | | | | |

| **Current and/or past depression treatment^a,b^** | | | | |
| --- | --- | --- | --- | --- |
|  | | Frequency | Percent | Valid percent |
| Valid | **Yes** | 14 | 38.9 | 45.2 |
|  | **No** | 17 | 47.2 | 54.8 |
|  | Total | 31 | 86.1 | 100.0 |
| Missing |  | 5 | 13.9 |  |
| Total | | 36 | 100.0 |  |
| a. Test population: **all patients with “current major depressive episode” based on the M.I.N.I.**  (the underlying questions refer to the last 2 weeks) | | | | |
| b. Past depression treatment: referring to the entire previous life | | | | |

| Offers of therapy (group without “current major depressive episode“)  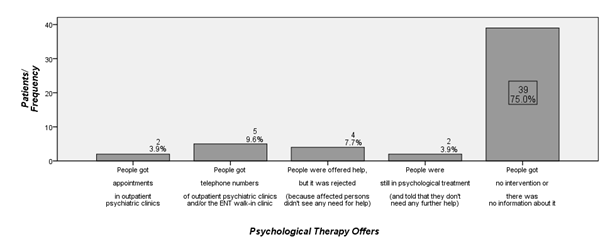 |
| --- |
| Test population: **all patients without current major depressive disorders (using M.I.N.I.); N = 52**  (the underlying questions refer to the last 2 weeks)  Please note: prescriptions of psychiatric drugs are not shown in the figure above.  Please note: if a patient was given an appointment in an outpatient psychiatric clinic, he/she was not listed again in one of the other categories (no double-counting) |

| **Current major depressive episode * Dry mouth cross-tabulation^a^** | | | | | | | |
| --- | --- | --- | --- | --- | --- | --- | --- |
|  | | | **Dry mouth^b^** | | | | **Total** |
|  |  |  | **Not**  **at all** | **A little** | **Quite**  **a bit** | **Very much** |  |
| **Current**  **major depressive episode^c^** | **Yes** | Count | 4 | 7 | 16 | 9 | 36 |
|  |  | % within current major depressive episode | 11.1% | 19.4% | 44.4% | 25.0% | 100.0% |
|  | **No^d^** | Count | 118 | 91 | 86 | 81 | 376 |
|  |  | % within current major depressive episode | 31.4% | 24.2% | 22.9% | 21.5% | 100.0% |
| Total | | Count | 122 | 98 | 102 | 90 | 412 |
|  |  | % within current major depressive episode | 29.6% | 23.8% | 24.8% | 21.8% | 100.0% |
| a. Test population: **total study group** (valid values) | | | | | | | |
| b. The item refers to the last week | | | | | | | |
| c. The underlying questions refer to the last 2 weeks | | | | | | | |
| d. No current major depression episode (M.I.N.I.) or WHO-5 sum score of ≥ 13 | | | | | | | |

| **Current major depressive episode * Trouble doing strenuous activities cross-tabulation^a^** | | | | | | | |
| --- | --- | --- | --- | --- | --- | --- | --- |
|  | | | **Trouble doing strenuous activities^b^** | | | | **Total** |
|  |  |  | **Not**  **at all** | **A little** | **Quite**  **a bit** | **Very much** |  |
| **Current**  **major depressive episode^c^** | **Yes** | Count | 3 | 4 | 15 | 14 | 36 |
|  |  | % within current major depressive episode | 8.3% | 11.1% | 41.7% | 38.9% | 100.0% |
|  | **No^d^** | Count | 115 | 105 | 105 | 52 | 377 |
|  |  | % within current major depressive episode | 30.5% | 27.9% | 27.9% | 13.8% | 100.0% |
| Total | | Count | 118 | 109 | 120 | 66 | 413 |
|  |  | % within current major depressive episode | 28.6% | 26.4% | 29.1% | 16.0% | 100.0% |
| a. Test population: **total study group** (valid values) | | | | | | | |
| b. The item does not refer to a specific time period | | | | | | | |
| c. The underlying questions refer to the last 2 weeks | | | | | | | |
| d. No current major depression episode (M.I.N.I.) or WHO-5 sum score of ≥ 13 | | | | | | | |

| **Current major depressive episode * Trouble taking a long walk cross-tabulation^a^** | | | | | | | |
| --- | --- | --- | --- | --- | --- | --- | --- |
|  | | | **Trouble taking a long walk^b^** | | | | **Total** |
|  |  |  | **Not**  **at all** | **A little** | **Quite**  **a bit** | **Very much** |  |
| **Current**  **major depressive episode^c^** | **Yes** | Count | 5 | 9 | 8 | 14 | 36 |
|  |  | % within current major depressive episode | 13.9% | 25.0% | 22.2% | 38.9% | 100.0% |
|  | **No^d^** | Count | 153 | 95 | 85 | 45 | 378 |
|  |  | % within current major depressive episode | 40.5% | 25.1% | 22.5% | 11.9% | 100.0% |
| Total | | Count | 158 | 104 | 93 | 59 | 414 |
|  |  | % within current major depressive episode | 38.2% | 25.1% | 22.5% | 14.3% | 100.0% |
| a. Test population: **total study group** (valid values) | | | | | | | |
| b. The item does not refer to a specific time period | | | | | | | |
| c. The underlying questions refer to the last 2 weeks | | | | | | | |
| d. No current major depression episode (M.I.N.I.) or WHO-5 sum score of ≥ 13 | | | | | | | |

| **Current major depressive episode * Worry cross-tabulation^a^** | | | | | | | |
| --- | --- | --- | --- | --- | --- | --- | --- |
|  | | | **Worry^b^** | | | | **Total** |
|  |  |  | **Not**  **at all** | **A little** | **Quite**  **a bit** | **Very much** |  |
| **Current**  **major depressive episode^c^** | **Yes** | Count | 2 | 1 | 18 | 14 | 35 |
|  |  | % within current major depressive episode | 5.7% | 2.9% | 51.4% | 40.0% | 100.0% |
|  | **No^d^** | Count | 122 | 147 | 75 | 33 | 377 |
|  |  | % within current major depressive episode | 32.4% | 39.0% | 19.9% | 8.8% | 100.0% |
| Total | | Count | 124 | 148 | 93 | 47 | 412 |
|  |  | % within current major depressive episode | 30.1% | 35.9% | 22.6% | 11.4% | 100.0% |
| a. Test population: **total study group** (valid values) | | | | | | | |
| b. The item refers to the last week | | | | | | | |
| c. The underlying questions refer to the last 2 weeks | | | | | | | |
| d. No current major depression episode (M.I.N.I.) or WHO-5 sum score of ≥ 13 | | | | | | | |

| **Current major depressive episode * Less sexual enjoyment cross-tabulation^a^** | | | | | | | |
| --- | --- | --- | --- | --- | --- | --- | --- |
|  | | | **Less sexual enjoyment^b^** | | | | **Total** |
|  |  |  | **Not**  **at all** | **A little** | **Quite**  **a bit** | **Very much** |  |
| **Current**  **major depressive episode^c^** | **Yes** | Count | 3 | 4 | 8 | 16 | 31 |
|  |  | % within current major depressive episode | 9.7% | 12.9% | 25.8% | 51.6% | 100.0% |
|  | **No^d^** | Count | 147 | 78 | 56 | 42 | 323 |
|  |  | % within current major depressive episode | 45.5% | 24.1% | 17.3% | 13.0% | 100.0% |
| Total | | Count | 150 | 82 | 64 | 58 | 354 |
|  |  | % within current major depressive episode | 42.4% | 23.2% | 18.1% | 16.4% | 100.0% |
| a. Test population: **total study group** (valid values) | | | | | | | |
| b. The item refers to the last week | | | | | | | |
| c. The underlying questions refer to the last 2 weeks | | | | | | | |
| d. No current major depression episode (M.I.N.I.) or WHO-5 sum score of ≥ 13 | | | | | | | |

| **Current major depressive episode * Sticky saliva cross-tabulation^a^** | | | | | | | |
| --- | --- | --- | --- | --- | --- | --- | --- |
|  | | | **Sticky saliva^b^** | | | | **Total** |
|  |  |  | **Not**  **at all** | **A little** | **Quite**  **a bit** | **Very much** |  |
| **Current**  **major depressive episode^c^** | **Yes** | Count | 8 | 10 | 8 | 9 | 35 |
|  |  | % within current major depressive episode | 22.9% | 28.6% | 22.9% | 25.7% | 100.0% |
|  | **No^d^** | Count | 150 | 96 | 72 | 51 | 369 |
|  |  | % within current major depressive episode | 40.7% | 26.0% | 19.5% | 13.8% | 100.0% |
| Total | | Count | 158 | 106 | 80 | 60 | 404 |
|  |  | % within current major depressive episode | 39.1% | 26.2% | 19.8% | 14.9% | 100.0% |
| a. Test population: **total study group** (valid values) | | | | | | | |
| b. The item refers to the last week | | | | | | | |
| c. The underlying questions refer to the last 2 weeks | | | | | | | |
| d. No current major depression episode (M.I.N.I.) or WHO-5 sum score of ≥ 13 | | | | | | | |

| **Current major depressive episode * Cough cross-tabulation^a^** | | | | | | | |
| --- | --- | --- | --- | --- | --- | --- | --- |
|  | | | **Cough^b^** | | | | **Total** |
|  |  |  | **Not**  **at all** | **A little** | **Quite**  **a bit** | **Very much** |  |
| **Current**  **major depressive episode^c^** | **Yes** | Count | 6 | 8 | 15 | 7 | 36 |
|  |  | % within current major depressive episode | 16.7% | 22.2% | 41.7% | 19.4% | 100.0% |
|  | **No^d^** | Count | 118 | 136 | 86 | 37 | 377 |
|  |  | % within current major depressive episode | 31.3% | 36.1% | 22.8% | 9.8% | 100.0% |
| Total | | Count | 124 | 144 | 101 | 44 | 413 |
|  |  | % within current major depressive episode | 30.0% | 34.9% | 24.5% | 10.7% | 100.0% |
| a. Test population: **total study group** (valid values) | | | | | | | |
| b. The item refers to the last week | | | | | | | |
| c. The underlying questions refer to the last 2 weeks | | | | | | | |
| d. No current major depression episode (M.I.N.I.) or WHO-5 sum score of ≥ 13 | | | | | | | |

| **Current major depressive episode * Less interest in sex cross-tabulation^a^** | | | | | | | |
| --- | --- | --- | --- | --- | --- | --- | --- |
|  | | | **Less interest in sex^b^** | | | | **Total** |
|  |  |  | **Not**  **at all** | **A little** | **Quite**  **a bit** | **Very much** |  |
| **Current**  **major depressive episode^c^** | **Yes** | Count | 4 | 4 | 8 | 16 | 32 |
|  |  | % within current major depressive episode | 12.5% | 12.5% | 25.0% | 50.0% | 100.0% |
|  | **No^d^** | Count | 159 | 79 | 55 | 39 | 332 |
|  |  | % within current major depressive episode | 47.9% | 23.8% | 16.6% | 11.7% | 100.0% |
| Total | | Count | 163 | 83 | 63 | 55 | 364 |
|  |  | % within current major depressive episode | 44.8% | 22.8% | 17.3% | 15.1% | 100.0% |
| a. Test population: **total study group** (valid values) | | | | | | | |
| b. The item refers to the last week | | | | | | | |
| c. The underlying questions refer to the last 2 weeks | | | | | | | |
| d. No current major depression episode (M.I.N.I.) or WHO-5 sum score of ≥ 13 | | | | | | | |

| **Current major depressive episode * Felt tired cross-tabulation^a^** | | | | | | | |
| --- | --- | --- | --- | --- | --- | --- | --- |
|  | | | **Felt tired^b^** | | | | **Total** |
|  |  |  | **Not**  **at all** | **A little** | **Quite**  **a bit** | **Very much** |  |
| **Current**  **major depressive episode^c^** | **Yes** | Count | 1 | 4 | 15 | 16 | 36 |
|  |  | % within current major depressive episode | 2.8% | 11.1% | 41.7% | 44.4% | 100.0% |
|  | **No^d^** | Count | 112 | 165 | 83 | 15 | 375 |
|  |  | % within current major depressive episode | 29.9% | 44.0% | 22.1% | 4.0% | 100.0% |
| Total | | Count | 113 | 169 | 98 | 31 | 411 |
|  |  | % within current major depressive episode | 27.5% | 41.1% | 23.8% | 7.5% | 100.0% |
| a. Test population: **total study group** (valid values) | | | | | | | |
| b. The item refers to the last week | | | | | | | |
| c. The underlying questions refer to the last 2 weeks | | | | | | | |
| d. No current major depression episode (M.I.N.I.) or WHO-5 sum score of ≥ 13 | | | | | | | |

| **Current major depressive episode * Felt weak cross-tabulation^a^** | | | | | | | |
| --- | --- | --- | --- | --- | --- | --- | --- |
|  | | | **Felt weak^b^** | | | | **Total** |
|  |  |  | **Not**  **at all** | **A little** | **Quite**  **a bit** | **Very much** |  |
| **Current**  **major depressive episode^c^** | **Yes** | Count | 0 | 5 | 15 | 15 | 35 |
|  |  | % within current major depressive episode | 0.0% | 14.3% | 42.9% | 42.9% | 100.0% |
|  | **No^d^** | Count | 153 | 136 | 75 | 17 | 381 |
|  |  | % within current major depressive episode | 40.2% | 35.7% | 19.7% | 4.5% | 100.0% |
| Total | | Count | 153 | 141 | 90 | 32 | 416 |
|  |  | % within current major depressive episode | 36.8% | 33.9% | 21.6% | 7.7% | 100.0% |
| a. Test population: **total study group** (valid values) | | | | | | | |
| b. The item refers to the last week | | | | | | | |
| c. The underlying questions refer to the last 2 weeks | | | | | | | |
| d. No current major depression episode (M.I.N.I.) or WHO-5 sum score of ≥ 13 | | | | | | | |

| **Time (full months) since first diagnosis of HNC^a,b,c,d^** | | | | |
| --- | --- | --- | --- | --- |
|  | N | Minimum | Maximum | Mean |
| **Time (full months) since first diagnosis of HNC** | 444 | 3 | 499 | 66.80 |
| a. Test population: **total study group**  (missing items: 9) | | | | |
| b. Only initial diagnoses of ENT tumors were taken into account (no other tumor entities) | | | | |
| c. Time between initial diagnosis of ENT tumor and questionnaire | | | | |
| d. Initial diagnosis of ENT tumor  - Panendoscopy or first verifiable therapy  - We always chose the first of the month  - If only a year was given, we chose the month of June (first of the month) | | | | |

| **Time (full months) since first diagnosis of HNC^a,b,c,d^** | | | | | |
| --- | --- | --- | --- | --- | --- |
|  | | Frequency | Percent | Valid Percent | Cumulative Percent |
| **Time (full months) since first diagnosis of HNC** | **≤ 12** | 72 | 15.9 | 16.2 | 16.2 |
|  | **13 - 24** | 64 | 14.1 | 14.4 | 30.6 |
|  | **25 - 36** | 40 | 8.8 | 9.0 | 39.6 |
|  | **37 - 48** | 43 | 9.5 | 9.7 | 49.3 |
|  | **49 - 60** | 38 | 8.4 | 8.6 | 57.9 |
|  | **≥ 61** | 187 | 41.3 | 42.1 | 100.0 |
|  | **Total** | 444 | 98.0 | 100.0 |  |
| Missing | System | 9 | 2.0 |  |  |
| Total | | 453 | 100.0 |  |  |
| a. Test population: **total study group**  (missing items: 9) | | | | | |
| b. Only initial diagnoses of ENT tumors were taken into account (no other tumor entities) | | | | | |
| c. Time between initial diagnosis of ENT tumor and questionnaire | | | | | |
| d. Initial diagnosis of ENT tumor  - Panendoscopy or first verifiable therapy  - We always chose the first of the month  - If only a year was given, we chose the month of June (first of the month) | | | | | |
